# Compartment Syndrome of the Pediatric Upper Extremity: Current Concepts

**DOI:** 10.3390/children12121696

**Published:** 2025-12-16

**Authors:** Thomas G. Pallan, Jackson T. Elkins, Alexandra M. Dunham, Julio J. Jauregui, Joshua M. Abzug

**Affiliations:** Department of Orthopaedics, University of Maryland School of Medicine, Baltimore, MD 21201, USA

**Keywords:** pediatrics, compartment syndrome, fasciotomy, upper extremity, fracture, neonatal, pressure, adolescent

## Abstract

**Highlights:**

**What are the main findings?**

- Pediatric upper extremity compartment syndrome is rare but often diagnosed late due to subtle signs and communication challenges, especially in young children and neonates.
- Despite delayed diagnosis and intervention, including delayed fasciotomy, children generally experience > 80% full functional recovery in most series, though evidence is primarily from lower extremity or mixed cohorts, with less data specific to the upper extremity.

**What are the implications of the main findings?**

- Current diagnostic and treatment protocols lack consensus and are often based on adult data or clinical dogma, highlighting the need for pediatric-specific guidelines.
- The pediatric population’s superior tissue healing and nerve regeneration suggest that delayed intervention and serial wound closure attempts may be more successful than in adults, though this is based on limited data, and early identification and treatment are still the main goals to prevent underestimating the urgency of treating compartment syndrome.

**Abstract:**

Pediatric compartment syndrome of the upper extremity is a rare but urgent surgical condition requiring timely diagnosis and intervention to prevent permanent damage. This review synthesizes current evidence on epidemiology, clinical presentation, diagnostic challenges, and management strategies. Despite diagnostic delays and variability in treatment timing including delayed fasciotomy, functional outcomes in children are generally favorable with >80% full functional recovery in most series. However, significant gaps remain in evidence-based guidelines, with the most critical being risk factors, diagnostic thresholds, and optimal wound closure techniques.

## 1. Introduction

Pediatric compartment syndrome of the upper extremity is a surgical emergency characterized by elevated intracompartmental pressure that compromises tissue perfusion, potentially leading to irreversible muscle and nerve damage if left untreated [1,2]. Pediatric cases often arise from diverse etiologies, including fractures, soft tissue injuries, vascular injuries, infection, or iatrogenic causes, such as IV infiltrations [3,4,5,6,7]. Diagnosis in the upper extremity is frequently delayed compared to other regions [4], potentially due to more subtle signs and diagnostic uncertainty [8,9]. Prompt recognition and fasciotomy are critical to prevent long-term sequelae like Volkmann’s ischemic contracture [5]. Yet, much of the current approach to diagnosis and management has no consensus guidelines available [10]. How the pressure level for diagnosing compartment syndrome in the upper extremity in children differs from that in adults, and the optimal strategy for fasciotomy wound management, including the timing and method of delayed closure, are among the most controversial aspects of diagnosis and management. Despite these gaps, functional outcomes in children are generally favorable. The objective of this narrative review is to summarize current knowledge regarding the pathophysiology, clinical presentation, diagnostic considerations, and management principles specific to pediatric upper-extremity compartment syndrome while highlighting areas where evidence remains limited and further study is needed.

## 2. Epidemiology & Etiology

Clinicians should be familiar with the various risk factors leading to upper extremity compartment syndrome to make an efficient and accurate diagnosis. The epidemiology of pediatric upper extremity compartment syndrome has been elucidated by a recent, large-scale national analysis [3]. Among 76,216 pediatric patients presenting to U.S. trauma centers with upper extremity long bone fractures, the incidence of compartment syndrome was 0.16% (*n* = 120) [3]. This figure is lower than reported in previous, smaller studies [11], which the authors attribute to the larger population and more diverse inclusion of fracture patterns and injury mechanisms in their analysis [3]. Upper extremity compartment syndrome is less common than lower extremity compartment syndrome, constituting 28% of all compartment syndrome cases in pediatric patients with long bone fractures [3]. Regarding the mechanism of injury, the vast majority of patients with upper extremity fractures sustained blunt trauma (73%), with falls being the most frequent specific mechanism (42%) [3]. Other mechanisms included motor vehicle collisions (7.5%), pedestrian strikes (0.8%), and motorcycle injuries (0.8%) [3]. However, on multivariable analysis, no specific high-energy mechanism was identified as an independent risk factor for developing compartment syndrome in the upper extremity, unlike in the lower extremity [3]. Demographically, the condition predominantly affects males, who comprised 79% of the upper extremity compartment syndrome cases, though male gender was not a statistically significant independent predictor on regression analysis [3]. Age is a critical factor, with adolescents at highest risk. The 14–17 year old age group accounted for 50% of all cases, and multivariable analysis confirmed that increasing age is an independent predictor [3]. The distribution of fracture sites for those with upper extremity compartment syndrome was: humerus (27%), ulna (13%), radius (10%), and concurrent radius/ulna (51%) [3]. The specific bone fractured is a major determinant of risk [3]. When compared to humerus fractures, isolated radial fractures (aOR 3.58), isolated ulnar fractures (aOR 2.68), and concurrent radial and ulnar (forearm) fractures (aOR 2.91) were all independent predictors, significantly increasing the odds of compartment syndrome [3]. Concerning fracture characteristics, patients with upper extremity compartment syndrome had a significantly higher prevalence of open fractures (41% vs. 10% in non-compartment syndrome) and comminuted fractures (22.0% vs. 4.7% in non-compartment syndrome) [3]. The presence of an open fracture (aOR 2.11), a comminuted fracture pattern (aOR 2.47), and an increasing number of fractures (aOR 1.84) were all identified as independent risk factors [3]. The modality of operative management also influences risk; patients undergoing open reduction internal fixation (ORIF) had significantly increased odds (aOR 2.85) of developing compartment syndrome, and every hour of delay in operative fixation was associated with a 0.4% increase in the adjusted odds of developing compartment syndrome [3]. This may be more reflective of the severity of injury rather than the intervention itself. Several important areas remain unexplored in including the specific risk associated with displaced fractures, the risk associated with fracture location (proximal, midshaft, distal), and a direct comparison of risk between different operative techniques such as plating versus nailing.

Unlike pediatric compartment syndrome associated with long bone fractures, there is a paucity of pediatric-specific data on hand compartment syndrome, likely attributable to its infrequency. In the sparse adult literature, traumatic etiologies predominate. A systematic review of 129 adult cases identified soft tissue injuries such as burns and crushes as the leading cause (86.8%), followed distantly by fractures (5.4%) and vascular insults (1.5%) [12]. Similarly, in a cohort of 53 adults undergoing hand fasciotomy, common causes included crush injuries (25%), prolonged decubitus (17%), and infections (11%). Additionally, 28% of cases resulted from hand fractures, and the dorsal interosseous compartment was involved in 83% of surgeries [13]. These adult patterns may offer some insight for pediatric hand compartment syndrome in high-risk scenarios like crush mechanisms or metacarpal fractures, but direct generalizability remains limited by age-related differences in tissue compliance, vascularity, and pressure thresholds. Given the near absence of pediatric data, future multicenter registries and prospective studies should specifically capture hand and shoulder involvement, which remain markedly underrepresented.

Neonatal compartment syndrome is exceedingly rare and typically presents either at birth or shortly thereafter in the immediate postpartum period [14]. The existing literature consists almost entirely of isolated case reports, with fewer than one hundred cases described to date, nearly all involving the upper extremity [15]. Common extrinsic factors include mechanical compression of the limb, which can occur during a difficult delivery, in multiparous pregnancies, when an umbilical loop is present, or due to an amniotic constriction band syndrome [1]. Intrinsic factors tend to be those that predispose the newborn to a hypercoagulable state such as sepsis and thrombophilic disorders [15,16,17]. The evidence is too sparse to reliably determine the relative frequency between intrinsic and extrinsic factors.

## 3. Diagnosis

### 3.1. Patient Assessment

The first step to clinically diagnosing pediatric acute compartment syndrome is to assess the patient’s condition. For adults and some older adolescents, “the 5 P’s” are used as signs of compartment syndrome: Pain out of proportion, Pallor, Paresthesia, Paralysis, and Pulselessness [2,18]. Since it is often difficult to reliably measure these factors in children, “the 3 A’s” are suggested as signs for pediatric compartment syndrome: Anxiety, Agitation, and Analgesia (increasing analgesia requirements) [2,18,19]. The 3 A’s may be especially helpful in preverbal children (generally <3 years), early–school-age children (approximately 3–7 years of age), and developmentally delayed children of any age who are often unable to reliably describe sensory changes or localize pain. Importantly, these clinical signs (“5 P’s” and “3 A’s”) are not mutually exclusive but rather complement one another.

### 3.2. Physical Examination

During the physical examination, any splints or casts should be removed, the skin should be observed for swelling or irregularities, compartments should be palpated for pain and discomfort, and relevant muscles should be stretched to identify tenseness [2,10]. Possible sensory and neurological deficits resulting from the patient’s injury create challenges to successfully complete physical examinations, especially in young children [2,7]. Importantly, decreased sensation and decreased motor function have also been associated with the diagnosis of late-stage compartment syndrome [2]. In patients with supracondylar fractures, Robertson et al. found that neurovascular injury was the greatest risk factor for developing compartment syndrome [7]. In extension-type supracondylar fractures, which comprise 98% of cases, anterior interosseous nerve (AIN) injury is most common followed by median nerve injury [20]. Median nerve involvement, especially complete palsy, should heighten suspicion more than isolated AIN involvement, which only involves motor function. This is due to combined sensory and motor loss with median nerve involvement, which can mask or complicate early compartment syndrome detection, and thus should prompt lower thresholds for serial neurovascular exams [20].

In our practice, we perform serial neurovascular and compartment examinations, as well as review of analgesia requirements, every 4 h during the first 24–48 h after injury, when the risk of compartment syndrome is greatest. During this period, we maintain elevation and close observation of the injured extremity, ensuring that the patient is not requiring increasing analgesic requirements and is not exhibiting anxiety or agitation that may reflect worsening pain. Assessment of motor and sensory function is performed at each evaluation to ensure that these aspects are unchanged or improving and not worsening. If examinations and symptoms remain stable throughout this initial 24–48 h window following the injury, the likelihood of evolving compartment syndrome becomes very low, and continued intensive monitoring is generally unnecessary.

### 3.3. Pressure Measurements

While the diagnosis of compartment syndrome is primarily clinical, relying on a thorough patient examination and serial assessments, pressure measurements can serve as an adjunct diagnostic tool in specific scenarios where the clinical exam is unreliable or unavailable. These include unconscious, uncooperative, or developmentally challenged patients, or in those with significant upper extremity injury accompanied by sensory or motor deficits [2,10,21,22]. Pressure measurements should not be used in isolation if possible. Absolute interstitial tissue pressures above 30 mmHg have traditionally been accepted as the threshold for compartment syndrome, and some authors have advocated using perfusion pressures (diastolic blood pressure minus compartment pressure) of <30 mmHg—or <40 mmHg in traumatized tissues—as an indication for fasciotomy [1,2,10,21,22]. Staudt et al. found that normal baseline compartment pressures in children are higher than in adults, though these findings were limited to the leg and may not directly extrapolate to the upper extremity [23]. Additionally, these findings have not translated into higher accepted thresholds for indication of fasciotomy [10].

Pressure instrumentation should be inserted into the suspected compartments, as determined by a physical exam, as well as surrounding compartments [24]. When measurements yield equivocal values, clinical judgement is key, and increased frequency of the serial exams should be performed to more closely monitor the patient.

### 3.4. Emerging Diagnostic Tools

In place of compartment pressure measurements, several studies have connected muscle oxygenation levels and compartment pressures [25,26,27,28]. This method is non-invasive and involves the use of Near-Infrared spectroscopy to accurately measure oxygenated and deoxygenated hemoglobin. Although some case studies support the use of Near-Infrared spectroscopy to diagnose compartment syndrome, much of the current literature focuses on adults and lower extremity injuries [25,26,27,28]. Another study explored the use of polarographic oxygen probes to directly measure oxygen levels in muscles, although tests have primarily been performed on animals [27]. Further studies are needed to test the reliability of Near-Infrared spectroscopy in pediatric cases of compartment syndrome. These measurement devices, at this time, are not widely available across care centers in the U.S.

### 3.5. Silent Compartment Syndrome

Silent compartment syndrome (SCS) refers to compartment syndrome without significant pain while at rest or with passive motion [29,30,31]. One of the defining characteristic symptoms of compartment syndrome is abnormally high levels of pain in the affected area [1,2]. If a patient is not experiencing pain, it can be incredibly difficult to correctly identify compartment syndrome and could lead to a delay in diagnosis and treatment. In the absence of pain, several case studies emphasize the importance of observations of firm compartments and high compartment pressure readings when available [29,30,31]. None of the case studies were able to determine an exact reason for the lack of pain. One found nerve injuries in 4 out of their 5 patients, pointing to possible sensory deficits as a cause [31]. Another case study suggested that pediatric patients may have a higher tolerance for elevated compartment pressures, since children typically exhibit higher average compartmental pressures than adults [30]. Treatment does not differ between those with SCS compared to those with traditional compartment syndrome [29,30,31]. Although silent compartment syndrome is rare and reported primarily through case series, its recurring documentation in pediatric patients highlights the importance of clinician vigilance.

### 3.6. Challenges with Diagnosing Pediatric Populations

Diagnosing practices in compartment syndrome differ between adult and pediatric populations as children often have difficulty communicating symptoms they are experiencing. Delays in the diagnosis of compartment syndrome in children are also common, especially in infants [32,33]. In a study with 233 children presenting with acute compartment syndrome, the mean time from injury to diagnosis was 22.0 h [33]. In comparison, a study including 16 infants with compartment syndrome experienced an average time to diagnosis of 31.1 h [32]. These delays in diagnosis in younger populations are most likely due to infants and toddlers’ lack of communication and cooperation [32]. For preverbal children, physicians may have to rely on caregiver observations alongside clinical cues to make proper diagnoses. Despite delays in presentation and diagnosis, outcomes in children are generally still favorable, with most achieving full functional recovery [22,32,33]. Despite this, timely and efficient treatment of compartment syndrome is still the goal to avoid tissue necrosis and neurovascular compromise. Consequently, extra care and preventative measures should be taken with younger at-risk populations.

### 3.7. Neonatal Compartment Syndrome

Neonatal compartment syndrome typically presents differently compared to older pediatric patients and adults. Nearly all reported neonatal cases involved the upper extremities, and the hallmark of the condition is the presence of sentinel skin changes, such as desquamation, blister/bullae formation, and skin necrosis [14,15,34]. Because normal intracompartmental pressure values have not been defined for neonates, the diagnostic value of pressure measurement in this population is uncertain, and management decisions are therefore exclusively based on clinical judgment [35].

## 4. Pathophysiology

The pathophysiology of pediatric compartment syndrome is thought to follow the same general principles described in adults, although the pediatric-specific evidence base remains limited [1,2]. A study measuring the normal compartmental pressure in adult forearms found that the mean compartmental pressures are 4.7 mmHg, 4.9 mmHg, and 3.6 mmHg for the volar, dorsal, and lateral compartments, respectively [36]. Pediatric forearm compartments tend to have higher baseline pressures, with one study finding the mean superficial volar compartmental pressure is 9.66 mmHg, the mean deep volar compartment is 10.22 mmHg, and the mean dorsal compartment is 12.90 mmHg [37]. These higher baseline pressures in children likely contribute to their greater tolerance to compartmental pressures, and may partly explain why adult compartmental thresholds do not translate directly to pediatric patients. If this compartmental pressure increases above venous pressure, vein wall collapse could result in venous congestion and reduced blood flow due to decreased pressure gradient with arterioles. Arteriole collapse and tissue hypoxia occur after tissue pressure reaches 30–40 mmHg, and tissue perfusion stops completely when pressures equal diastolic blood pressure. If decompression occurs quickly, cells will return to their original morphology and function in a week. If pressure is not released, subsequent cell death will release factors that initiate additional cellular damage, inflammatory responses, ischemia, and more tissue pressure in a positive feedback loop [1].

The pathophysiology of neurological dysfunction during compartment syndrome is similar to muscle; as pressures increase, local perfusion is inhibited, which may result in nerve ischemia [38]. One study by Gelberman et al. found that when adult forearm compartments were compressed to 50 mmHg, the sensory nerve action potential (SNAP) was absent within 25–40 min of compression and the compound muscle action potential (CMAP) amplitude decreased by 66–100% [39]. Above 60 mmHg, SNAP amplitude was absent within 10–40 min and CMAP amplitude decreased by 71–100% [39]. Further studies are needed to determine the effects of pediatric compartment syndrome on neurological function. Much of the pathophysiologic evidence in the literature is derived from adult or animal models, so clinicians must exercise caution when extrapolating the data to children.

## 5. Management & Outcomes

### 5.1. Timing

The time window before pediatric patients with upper extremity compartment syndrome experience irreversible muscle and nerve damage remains uncertain. Studies on adult patients have generally found that permanent myonecrosis occurs after 8 h of no perfusion, with some studies finding irreversible tissue damage emerging as early as 3 h [40,41,42,43]. However, a meta-analysis by Lin et al. reported that 85% of patients had a full recovery after undergoing a fasciotomy at an average time of 25.4 h after injury [33]. Lin et al. also reported that the interval between injury and fasciotomy did not differ significantly between patients who regained full function and those who failed to do so [33]. In their most recent case series, 100% of pediatric patients (*n* = 21) achieved full functional recovery after decompressive fasciotomy for acute compartment syndrome, including all 8 patients who were treated in a delayed fashion (≥24 h after injury), suggesting that the effective treatment window in children may be longer than in adults [22]. Nevertheless, studies specific to the upper extremity are still needed to define this timeframe precisely.

Even in delayed presentations, children demonstrate remarkable tissue recovery [33]. As such, overly aggressive removal of muscle that appears nonviable at the initial fasciotomy should be avoided, since such tissue may still revascularize and recover function with time [18,44,45]. Additionally, children exhibit remarkable nerve regeneration capacity [46]. In Jerome’s series of 24 pediatric patients with upper extremity compartment syndrome, even those presenting late, some up to 6 days post-injury, derived significant benefit from fasciotomy, with 67% of the delayed subgroup achieving full functional recovery [46]. In this series, neurolysis of the median and ulnar nerves from the carpal tunnel to the cubital tunnel was performed in delayed cases where severe nerve entrapment was identified intraoperatively. Taken together, while children may tolerate longer delays than adults, early recognition and prompt fasciotomy remain the standard goal to minimize the risk of irreversible injury.

Unlike older children, neonates appear to have a much narrower therapeutic window. Ragland et al. reported a series of 24 cases of neonatal compartment syndrome, in which only one infant underwent fasciotomy within the first 24 h of life—and notably, this was the only patient who did not experience subsequent complications [14]. Clinicians should treat these cases as true surgical emergencies, with an even greater sense of urgency than in older children.

### 5.2. Initial (Preoperative) Management

To reduce swelling, any constrictive dressing/casting should be removed, and the extremity should be elevated. Given that pain is one of the most common symptoms of acute compartment syndrome, an analgesic may be given. However, there is uncertainty surrounding the optimal analgesic regimen that maintains diagnostic reliability, as there are concerns that the analgesic may mask symptoms. Increasing analgesia requirements are associated with pediatric and adult compartment syndrome [11,47]. Current adult anesthesia literature emphasizes that regional anesthesia, when used judiciously, does not inherently delay or obscure the diagnosis of compartment syndrome [48,49]. Lam et al. reviewed the evidence across peripheral nerve blocks, epidural anesthesia, and systemic opioids, concluding that low-concentration, site-specific peripheral nerve blocks offer effective analgesia while generally preserving the ability to detect breakthrough ischemic pain [49]. In contrast, dense neuraxial anesthesia or opioid-based regimens (including patient-controlled analgesia) are more commonly associated with delayed recognition of compartment syndrome, underscoring the importance of dilute, short-acting regional techniques coupled with vigilant monitoring [49,50,51]. However, there are no studies specifically investigating this for upper extremity compartment syndrome in the pediatric population. Based on our experience, we suggest the following practical approach: low-concentration, site-specific peripheral nerve blocks are reasonable when paired with close monitoring and serial examinations, whereas dense neuraxial or PCA-heavy regimens should be used with caution in high-risk patients. Studies investigating the correlation between exam intervals and diagnostic delay rates to show whether more frequent exams truly improve outcomes may be warranted.

### 5.3. Indications and Contraindications for Fasciotomy

Once compartment syndrome is diagnosed in the upper extremity, there is no non-operative management, so diagnosis automatically indicates fasciotomy(ies). Unlike in adults, there is no clear timeframe that is too late to perform a fasciotomy in children [22,33]. While in adults, late fasciotomies are considered more detrimental than beneficial, with delayed intervention associated with higher complication and infection rates, pediatric data continue to support a relatively aggressive approach, with no clear cutoff beyond which intervention is futile [22,33,52,53,54]. Further studies are needed to determine this timeframe, but current sources recommend proceeding with fasciotomy(ies) even in delayed presentations [1,2].

### 5.4. Surgical Technique

Multiple surgical techniques for upper extremity fasciotomy have been described, varying by anatomic region. In general, patients are positioned supine and given general anesthesia and preoperative antibiotics, typically without a tourniquet. After opening the primary fascial envelope, the need for associated decompression is evaluated in soft-tissue structures, and possible sites of neurovascular constriction are considered for release. Following decompression, the skin is not closed to avoid possible re-elevation of compartmental pressures [1,2]. For the forearm, a long volar (S-shaped/curvilinear) incision is used and often sufficient to decompress all compartments, with a dorsal incision added only when pressures remain elevated in the dorsal and mobile wad compartments [10]. If only the mobile wad remains inadequately decompressed after the volar incision, a targeted incision directly over the mobile wad can be used instead of a full dorsal incision [10]. In the hand, all ten compartments are typically decompressed through two dorsal incisions over the second and fourth metacarpals, with additional thenar and hypothenar incisions made as needed for complete release [10]. For the upper arm, the anterior and posterior compartments are decompressed with a lateral incision on both sides; the incisions may be extended distally if the deltoid compartment needs decompression [1]. However, upper arm compartment syndrome is extremely rare, and the literature lacks extensive evidence for this surgical method [7,55,56]. One case study on a pediatric patient with compartment syndrome following a closed reduction and percutaneous pinning of a supracondylar fracture and one adult patient with compartment syndrome following humeral nailing reported positive results and no complications using the technique described [55,56]. Additional studies with larger sample sizes are needed to confirm the validity of this proposed upper arm fasciotomy method. During these procedures, when debriding necrotic tissue in a pediatric patient, special care should be taken to not be overly aggressive due to the potential for tissue recovery [18,44,45].

A minimally invasive “mini-fasciotomy” or “pie-crust” technique uses multiple small incisions to decompress affected compartments. In a pediatric series of 56 patients, Yuan et al. reported no postoperative infections, neurovascular injuries, osteomyelitis, or delayed union, and all patients achieved full sensory and functional recovery [57], though the study lacked significance testing or direct comparison groups. Hu et al. evaluated this method with vacuum-sealing drainage in 126 children and found significantly lower incision-infection rates and shorter hospital stays [58]. The primary limitation of this approach is the restricted exposure for debridement and neurovascular repair. Given its limited adoption and incomplete evidence base, this technique should be reserved for research settings or highly selected pediatric cases until further studies clarify its reproducibility, efficacy, and long-term outcomes. Current evidence continues to support traditional open incision approaches as the standard of care for upper extremity decompression, given the consistently low infection rates reported in the pediatric fasciotomy literature [33,59,60].

### 5.5. Post-Operative Wound Management

The management of fasciotomy wounds following compartment syndrome in the pediatric upper extremity remains largely understudied, with limited evidence primarily from retrospective case series. The literature is largely in agreement that primary closure is not recommended. While some have performed closure in the same surgery as the fasciotomy in the pediatric population [11], primary wound closure is generally discouraged, as it can limit visualization of potentially necrotic muscle tissue and elevate intracompartmental pressure, precipitating recurrent compartment syndrome [61,62,63,64]. Delayed primary closure (DPC) is generally preferred over split-thickness skin grafting (SG) when feasible. SG is associated with donor-site morbidity, does not preserve sensation at the fasciotomy site, and generally provides worse cosmetic and functional outcomes compared to DPC [65,66]. One study also showed that hospital stays were shorter following DPC (6–7 days) versus SG (11–12 days, *p* < 0.02), though this evidence was from an adult cohort [67]. While DPC does not fully mitigate cosmetic concerns, as upper-extremity scars remain a frequent source of dissatisfaction [60], SG is typically used only when multiple DPC attempts have failed or when the degree of tissue loss precludes closure.

The literature strongly supports the idea that DPC after fasciotomy for compartment syndrome appears to be significantly more feasible in children than in adults. In a cohort of 82 pediatric patients, including 35 (43%) upper extremity fractures, Rademacher et al. reported that 72% of fasciotomy wounds left open after the initial surgery were ultimately closed through DPC [68]. Even when wounds could not be closed at the first debridement, continued attempts at DPC across successive debridement remained safe with no increase in complication rates [68]. Compared with flap or skin graft coverage, DPC was associated with a shorter hospital stay (8 days vs. 12 days, *p* < 0.001) and no increase in complication rates (19% vs. 26%) [68]. These findings suggest that pediatric tissues possess greater healing potential and pliability, allowing for successful closure through serial management.

Most wounds can be closed in three attempts via DPC not including the initial fasciotomy [60,68,69], but if these attempts are not successful, there is disagreement about the number of times that serial debridement and DPC should be attempted before deferring to SG. Lim et al., in a retrospective review of 112 pediatric fasciotomy wounds, recommends moving to SG after the third DPC attempt, noting that 81.3% of wounds required grafting beyond that point [70]. In contrast, Rademacher et al. found that continued attempts at DPC across successive debridement remained safe and effective past the third attempt, with success rates of 50–67% through the third, fourth, and fifth attempts [68]. The differing success rates beyond the third DPC attempt between Lim et al. and Rademacher et al. may be the result of differences in injury severity, wound characteristics, and closure techniques. Based on our clinical experience, we recommend attempting DPC up to three times; if closure remains unsuccessful after three attempts, we favor proceeding to SG to minimize the risk of infection, prolonged hospitalization, and additional tissue trauma, while recognizing that select wounds may still be amenable to further DPC on a case-by-case basis.

No consensus exists on the optimal adjunctive technique for closure of DPC fasciotomy wounds in pediatric upper-extremity compartment syndrome. In a meta-analysis of 23 studies (739 fasciotomies, including 118 upper extremity cases overall), Jauregui et al. reported 92.4% DPC success (95% CI: 79.8–99.1%) using gradual suture approximation, outperforming negative pressure wound therapy (NPWT) at 78.1% (95% CI: 74.6–81.4%) [71]. Dynamic dermatotraction showed a comparably high success rate of 92.7% (95% CI: 85.1–97.7%), but it had the highest complication rate (18.40%) compared to NPWT (2.49%) and gradual suture approximation (14.83%) [71]. In a review by Kakagia, gradual suture approximation (22 studies, 332 patients) achieved delayed primary closure in 6 to 20 days, outperforming negative pressure wound therapy (7 studies, 567 patients) with mean durations ranging from 7 to 26 days, while dynamic dermatotraction (15 studies, 132 patients) required 1 to 15 days [62]. A summary of the techniques and their success rates is displayed in Table 1.

Delayed closure techniques can also be used in conjunction. Souder et al. recommends vessel-loop dermatotraction combined with NPWT as their preferred closure method for large, swollen fasciotomy wounds in children, noting that most such wounds achieve DPC and citing children’s increased capacity for tissue recovery after injury [10]. Taken together, these findings suggest that gradual suture approximation remains a practical first-line strategy, striking a good balance between closure success rate and complication rate, with NPWT or dynamic dermatotraction reserved for wounds with specific challenges. Further studies are needed specific to the pediatric upper extremity to more definitively determine the optimal adjunctive wound closure technique. Additionally, further perioperative questions also remain unanswered regarding the optimal interval timing for second-look surgery and wound closure, whether 24, 48, or 72 h yields the best outcomes, and there is likewise no established guidance on antibiotic prophylaxis intervals during serial returns to the operating room.

## 6. Summary

Pediatric upper-extremity compartment syndrome is a rare but urgent condition that presents unique diagnostic and management challenges. It most commonly occurs after forearm fractures, particularly concurrent radius and ulna injuries, and is more frequent in adolescents, males, and patients with open, comminuted, or multiple fractures. Delays in operative treatment of fractures or the presence of neurovascular injury further increase the risk, highlighting the need for clinicians to maintain a high index of suspicion in these situations. Diagnosis should be primarily clinical, relying on serial neurovascular examinations and signs such as anxiety, agitation, and escalating analgesic requirements, with compartment pressure measurements used when it is difficult to confirm a diagnosis and for specific patient populations. Neonatal compartment syndrome, while exceedingly rare, presents differently from older children. It often manifests as sentinel skin changes rather than classic pain or neurological deficits and often has worse outcomes than in older children.

Although children exhibit remarkable capacity for tissue and nerve recovery, timely recognition and treatment, including fasciotomy(ies), are still the primary goal. Traditional open fasciotomy remains standard, with minimally invasive approaches reported in limited series. Postoperative wound management favors DPC whenever feasible, with gradual suture approximation generally achieving the best balance between closure success and complication rates. Clinicians should reserve skin grafting for cases where closure is not possible after three attempts of DPC.

There is a relative paucity of pediatric-specific literature investigating upper-extremity compartment syndrome, and much of the current guidance is extrapolated from adult or lower-extremity data, which may not fully account for children’s unique physiology, pressure tolerances, and enhanced tissue healing. Clinicians should exercise caution when applying adult-based thresholds and protocols to pediatric patients. Overall, while children often achieve excellent outcomes, even with delayed intervention, early recognition, vigilant monitoring, and individualized wound management remain critical.

## 7. Future Directions

Many critical questions regarding pediatric upper extremity compartment syndrome remain unanswered. In terms of epidemiology and etiology, future research is needed to further clarify the risks associated with displaced fractures, how fracture site location influences risk, how different methods of surgical fixation, such as plating versus intramedullary nailing affect risk, and the incidence of compartment syndrome arising from hand and upper arm fractures. In neonates, prospective work is needed to better distinguish the relative contributions of intrinsic versus extrinsic factors leading to compartment syndrome. In terms of diagnosis, additional studies are needed to define baseline compartment pressures in children and neonates, including variation across individual upper arm, forearm, and hand compartments, to create accurate pediatric-specific pressure thresholds. Diagnostic research should also evaluate the sensitivity and specificity of tools such as near-infrared spectroscopy in pediatric patients. Further work is required to determine the safety and diagnostic reliability of various analgesic regimens, given that adult data may not translate to younger populations. With regard to timing, prospective studies are needed to elucidate when it becomes too late to perform a fasciotomy in a pediatric patient. Concerning surgical techniques, studies are needed to determine the efficacy of mini fasciotomy in comparison to the traditional open approach. In terms of wound care management, research is needed to determine optimal wound-closure strategies, including how many attempts at delayed primary closure should precede conversion to a skin graft and which adjunctive methods achieve the best outcomes. Finally, future studies should define the ideal timing between serial second-look surgeries after the index fasciotomy, whether returning at 24, 48, or 72 h yields better outcomes, and establish evidence-based guidance on appropriate antibiotic dosing intervals during these repeated attempts at delayed primary closure.

## Figures and Tables

**Table 1:** Summary of adjunctive technique for DPC based on Jauregui et al. and Kakagia.

Technique	DPC Success Rate	Complication Rate	Time to Closure
Gradual Suture Approximation	92.4%	14.8%	6–20 days
NPWT	78.1%	2.5%	7–26 days
Dynamic Dermatotraction	92.7%	18.4%	1–15 days

## Data Availability

No new data were created or analyzed in this study. Data sharing is not applicable to this article.

## Abbreviations

The following abbreviations are used in this manuscript:

DPC	Delayed primary closure
SG	Split-thickness skin grafting
SCS	Silent compartment syndrome
NPWT	Negative pressure wound therapy
